# Prognosis versus Actual Outcomes in Stereotactic Radiosurgery of Brain Metastases: Reliability of Common Prognostic Parameters and Indices

**DOI:** 10.3390/curroncol31040132

**Published:** 2024-03-26

**Authors:** Julian Mangesius, Thomas Seppi, Christoph Reinhold Arnold, Stephanie Mangesius, Johannes Kerschbaumer, Matthias Demetz, Danijela Minasch, Samuel Moritz Vorbach, Manuel Sarcletti, Peter Lukas, Meinhard Nevinny-Stickel, Ute Ganswindt

**Affiliations:** 1 Department of Radiation Oncology, Medical University of Innsbruck, 6020 Innsbruck, Austria; 2 Department of Neuroradiology, Medical University of Innsbruck, 6020 Innsbruck, Austria; 3 Department of Neurosurgery, Medical University of Innsbruck, 6020 Innsbruck, Austria

**Keywords:** radiosurgery, brain metastases, prognosis, prognostic scores, outcome, sex differences

## Abstract

This study aims to evaluate the clinical outcome of stereotactic radiosurgery as the sole treatment for brain metastases and to assess prognostic factors influencing survival. A total of 108 consecutive patients with 213 metastases were retrospectively analyzed. Treatment was determined with close-meshed MRI follow-up. Various prognostic factors were assessed, and several prognostic indices were compared regarding their reliability to estimate overall survival. Median overall survival was 15 months; one-year overall survival was 50.5%. Both one- and two-year local controls were 90.9%. The rate of new metastases after SRS was 49.1%. Multivariate analysis of prognostic factors revealed that the presence of extracranial metastases, male sex, lower KPI, and progressive extracranial disease were significant risk factors for decreased survival. Of all evaluated prognostic indices, the Basic Score for Brain Metastases (BSBMs) showed the best correlation with overall survival. A substantial survival advantage was found for female patients after SRS when compared to male patients (18 versus 9 months, *p* = 0.003). SRS of brain metastasis is a safe and effective treatment option when frequent monitoring for new metastases with MRI is performed. Common prognostic scores lack reliable estimation of survival times. Female sex should be considered as an additional independent positive prognostic factor influencing survival.

## 1. Introduction

Brain metastases (BMs) are the most frequent malignant intracranial lesions in adults, accounting for up to 40% of all intracranial tumors. Improvements in cancer therapy increasingly prolong patient survival and lead to an increased rate of BM [1,2]. While newer targeted therapies have become a viable treatment option for selected patients in recent years, surgical resection and radiotherapy represent the cornerstones of modern treatment [3]. Stereotactic radiosurgery (SRS) can provide high local control (LC) rates by delivering a large and highly conformal single radiation dose while sparing surrounding brain tissue. SRS has a favorable impact on neurocognition and quality of life in comparison to whole brain radiation therapy (WBRT). Therefore, SRS is the standard therapy option for single or limited-in-number, non-resectable BM [4]. Recent data increasingly show that survival after SRS as an initial treatment for up to 10 brain metastases is non-inferior compared to 2–4 BMs [5,6,7]. However, SRS also entails a significant risk of radiation necrosis (RN), previously reported to occur in 8–24% of treated metastases [8,9], which can be lowered by technical advances [10]. The diagnosis of RN, and especially the distinction from real tumor progression, is challenging but critical to evaluate therapy options [11,12]. While SRS alone delivers impressive LC rates, intracranial control and the time to appearance of new metastases are significantly reduced when compared to WBRT. Frequent monitoring for new BM is therefore imperative, in addition to conducting more studies, for the determination of dose tolerance parameters across a broad spectrum of patients.

In the present study, we report our experience with LINAC-based SRS in routinely treating BM, thereby dedicating special attention to the validation of relevant prognostic indices and independent factors.

## 2. Materials and Methods

Between August 2003 and March 2018, 108 consecutive patients underwent single fraction linear accelerator-based SRS without adjuvant WBRT as initial therapy for intracranial metastases. Patients were chosen for SRS treatment in case of synchronous or metachronous, singular or multiple brain metastases (up to 5 lesions), with a KPI of at least 6. Patients with large or symptomatic metastases were assigned to surgical resection unless unsuitable for surgery. 

All retrospectively analyzed patients received conformal SRS applying five to seven non-coplanar arcs with cone collimators. Head immobilization was performed either by using rigid stereotactic fixation, or non-invasively by multi-layered thermoplastic masks. Target volumes were determined by using planning CT scans together with fused contrast-enhanced T1-weighted MRI scans. GTV was expanded by a margin of 1 mm for frameless SRS, and no expansion was used for frame-based SRS. The median prescribed dose to the 80% isodose was 20 Gy and ranged from 15 to 22 Gy, depending on metastasis volume, location, proximity to vital structures, history of WBRT or previous SRS, and tumor histology. Patient position was corrected and verified by means of cone-beam CT verification prior to treatment. Position accuracy was corrected to <1 mm xyz translation and <1° rotation. 

Follow-up by contrast-enhanced MRI scans and clinical examination was performed every 3 months until death, or at any time if clinically indicated. Two neuroradiologists independently diagnosed local recurrence, pseudo-progression, and RN. Local progression was defined as increased radiologic volume not explainable by RN or pseudo-progression. In the case of indicated surgical resection, histologic analysis was used to distinguish local recurrence from RN. PTV size as a risk factor for local recurrence was evaluated. Toxicity was assessed according to the RTOG common toxicity criteria. 

Evaluation of 4 of the 108 consecutively SRS-treated BM patients was incomplete because of unavailable imaging within the 6 months before death. 

Overall survival (OS) was defined as the elapsed time from the first SRS treatment until death. All patients alive at the end of the study (N = 24, 22.6%) were censored on the day of the last follow-up. Distant intracranial control was defined as the elapsed time until the appearance of new intracranial metastases. 

Several known prognostic factors for survival, including KPS, age, status of systemic disease, primary tumor control, presence of extracranial metastases, number of intracranial metastases, volume of the treated metastases, and time from primary tumor diagnosis to CNS treatment, were assessed according to RPA [13], GPA [14], SIR [15], BSBM [16], and Rades et al [17]. All listed PIs were calculated and ranked regarding their prognostic reliability in our patient cohort. In addition, sex, prescribed dose, histology of primary tumors as well as diagnosis-specific molecular parameters and mutations (lung: EGFR, ALK; melanoma: BRAF; breast: ER/PR receptor status, HER2) were investigated regarding their potential as independent prognostic factors.

IBM SPSS Statistics 24 (IBM Cooperation, Armonk, NY, USA) was used for statistical analysis. OS, intracranial control, and local control were determined by Kaplan–Meier analysis; log-rank test was used to evaluate prognostic factors. The Cox proportional hazard model was applied for uni- and multivariate analyses of prognostic factors. Receiver Operating Characteristics (ROCs) were used to compare the reliability of PIs. A binominal logistic regression model (with ascertained linearity of continuous variables by the Box–Tidwell procedure) was used to assess whether prognostic factors might differ between sexes. This model was chosen to ensure that an unequal distribution of prognostic factor values between sex groups did not bias our analysis. 

## 3. Results

A total of 108 patients (213 metastases) were analyzed. The patient characteristics are presented in Table 1. The median follow-up was 11 months (range: 0–99 months). The median tumor volume was 0.53 ccm and ranged from 0.04 to 13.29 ccm. 

### 3.1. Outcomes

**Figure 1:**
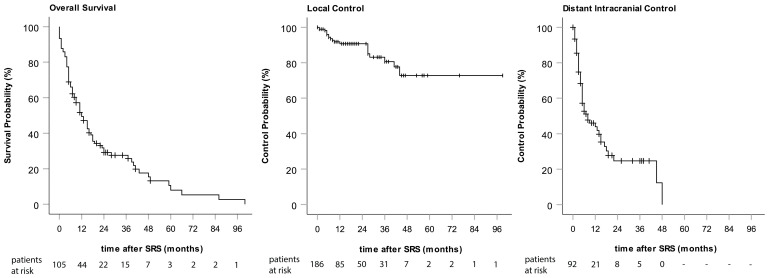
Kaplan–Meier curves of overall survival, local control, and distant intracranial control.

The outcome data are shown in Figure 1. The median OS after SRS was 15 months. The overall rate of patients surviving 1 and 2 years was 50.5% and 32.5%, respectively. A total of 8.1% of the investigated patients survived more than 5 years after SRS. A substantial difference in OS was observed depending on the type of primary tumor, which was statistically significant (*p* < 0.001). Of all BM histologies, breast cancer (N = 11) had the best prognosis, with a median OS of 24 months. The median OS was 17 months for NSCLC adenocarcinoma (N = 40), 6 months for NSCLC squamous cell carcinoma (N = 9), 11 months for RCC (N = 9), and 9 months for melanoma (N = 20).

An overall LC of 89.8% was achieved by SRS of BMs. In total, only 19 metastases recurred locally. One- and two-year LC rates were 90.7% (Figure 1). A PTV size of larger than 1 ccm correlated with a higher risk for local recurrence (Pearson Chi-Square X^2^(1) = 6.565 *p* = 0.01). However, PTV size had no influence on OS. Metastases were surgically removed after SRS in the cases of six suspected local recurrences: two of these turned out to represent RN, whereas four were confirmed to be local recurrences. 

A total of 49.1% of our patients developed new BMs after SRS. Of these, 39.6% received WBRT (21.5% of the total). The median time from the first SRS to the appearance of new metastases was 9 months. The one- and two-year distant intracranial control rates were 47.4% and 28.3%, respectively (Figure 1). 

Of all treated metastases, 37 (19.4%) showed evidence of either RN or pseudo-progression. Medium time to RN was 7 months, and the latest onset of RN was 41 months after SRS. Diagnosis of RN had no significant impact on OS. A PTV of >1 ccm was significantly correlated with the occurrence of RN (*p* = 0.021). 

### 3.2. Prognostic Factor Evaluation

Univariate analysis of prognostic factors showed that the presence of extracranial metastases, male sex, a lower KPI, and progressive systemic disease status were significant risk factors for impaired survival. Of these factors, only male sex, lower KPI, and progressive systemic disease were also significant in poor OS prognosis when analyzed by multivariate Cox proportional hazards regression (Table 2). Diagnosis-specific molecular markers (EGFR, ALK, BRAF, HER2, and ER/PR) showed no significant impact on the outcome of SRS.

### 3.3. Evaluation of Prognostic Indices

**Figure 2:**
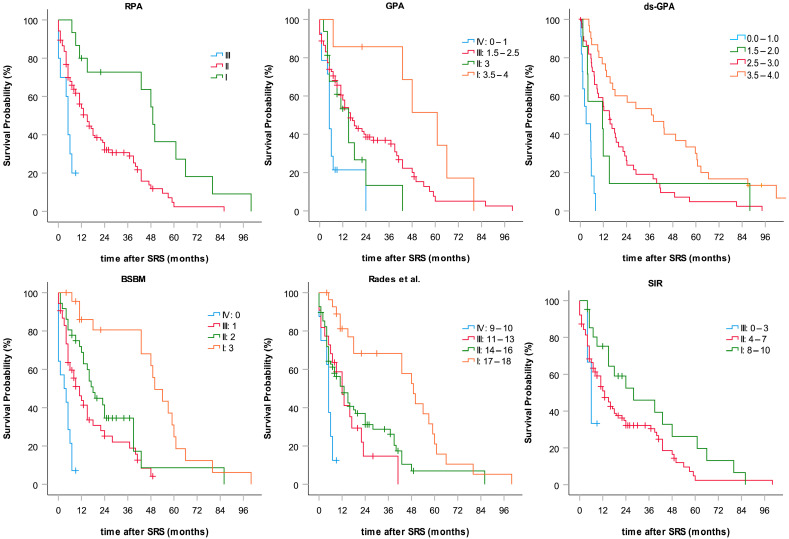
Subgroup-categorized Kaplan–Meier curves of overall survival calculated for six common prognostic indices.

OS data related to BSBM, GPA, RPA, SIR, and the scoring of Rades et al. are shown in Table 3 and Figure 2. With the exception of SIR, all prognostic indices were significantly associated with survival when applied to our cohort of patients. 

**Figure 3:**
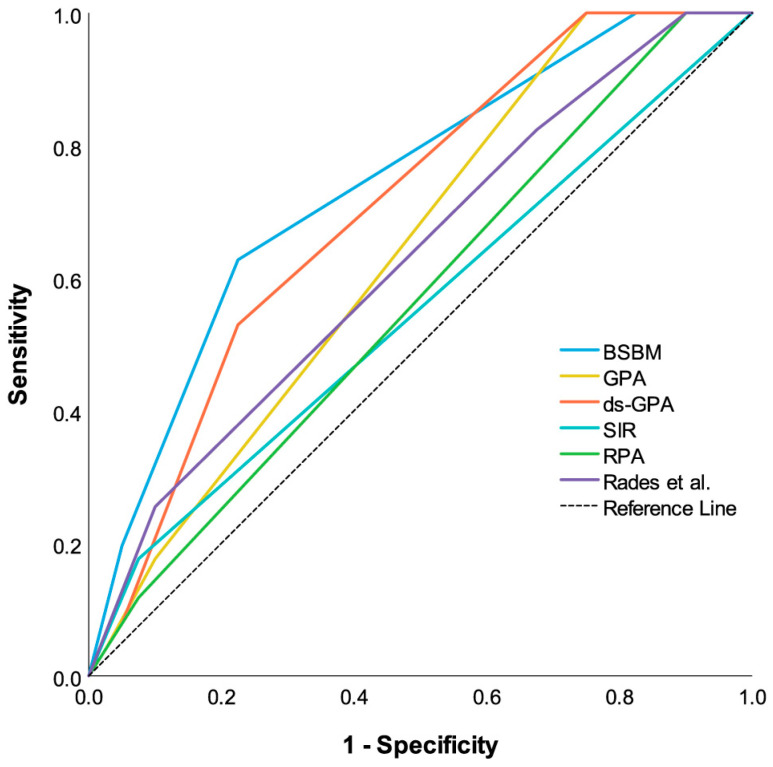
Comparative receiver operating characteristic curves (ROCCs) for each prognostic index estimating one-year survival probability after SRS. Predictive power of each PI is indicated by area under the curve of ROCC. Larger area under the curve indicates greater prognostic potential.

Using Receiver Operating Characteristics (ROC) analysis to evaluate the accuracy of one-year OS prediction, the best reliability was found for BSBM (area under the curve (AUC) = 0.75, *p* < 0.001), followed by the diagnosis-specific GPA (AUC = 0.71, *p* = 0.001), the original GPA (AUC = 0.64, *p* = 0.022), the score of Rades et al. (AUC = 0.62, *p* = 0.037), and RPA (AUC = 0.56, *p* = 0.28). SIR was determined as the least discriminative (AUC = 0.55, *p* = 0.40) (Figure 3). 

### 3.4. Sex and OS

Regarding their OS, female patients exhibited a markedly increased survival compared to males (median OS: 18 versus 9 months). The sex-related difference in OS was significant (log-rank X^2^(1) = 7.878, *p* = 0.005). This finding is still valid after the exclusion of patients affected by mainly sex-specific breast-cancer-derived BMs from comparative statistical analysis (log-rank X^2^(1) = 5.728, *p* = 0.017). To ascertain sex as an independent prognostic factor, a binomial logistic regression model was used to analyze and rule out all other investigated prognostic factors, which might potentially differ between male and female patients. None of the investigated prognostic factors differed significantly between men and women, except for the number of BMs, i.e. female patients were affected by even more metastases than male patients (mean male = 1.4; mean female = 1.8; *p* = 0.01).

## 4. Discussion

Our reported OS data (median OS = 15 months; 1-year OS rate = 50.5%) are comparable to other studies investigating SRS as the sole treatment for BMs, which show a median OS of 8-15 months [8,18,19,20,21,22,23]. In our single-center study with 108 patients treated for 1–5 BMs, the overall LC was 89.7%, with both a 1- and 2-year LC rate of 90.9%. Lutterbach et al. [24] reported a 1-year LC of 91% after SRS alone in a cohort of 101 patients, who suffered from 1–3 BMs. Minniti et al. [9] demonstrated similar results in 1-year and 2-year LC rates (92% and 84%, with 206 patients). Applying SRS alone in 153 patients, Pirzkall et al. [25] found an LC rate of 89% and 72% after one and two years of follow-up, respectively. LC after two years in the SRS-only arm of the EORTC 22952-26001 study [8] was 69%. In an analogous work, Brown et al. [23] reported a 1-year LC of 72.8% for 111 patients. Concerning former reports, our OS and LC data are highly comparable to studies with the lowest local recurrence and longest OS rates. 

While LC correlates with appropriate dose delivery [26], the risk of RN rises with elevated prescribed doses and greater target volumes [27,28,29]. Wiggenraad et al. [30] systematically reviewed that a dose delivery of less than 20 Gy severely impairs LC. Bohoudi et al. [28] proposed a method for isotoxic SRS planning based on V_12Gy_, a known predictor for RN. In the present study, a dose of 20 Gy to the tumor outline was pursued if permitted by size and location. As a result, an acceptable 19.4% of all treated metastases developed radiographic evidence for RN (grades 1–3). Our finding is congruent with comparable studies reporting on RN rates (grades 1–3), ranging from 12.1% up to 24% when applying similar dose concepts [9,10]. 

The debate is ongoing on whether it is advantageous to combine WBRT and SRS. Studies have shown that adding WBRT can improve local and distant intracranial control and delay the appearance of new metastases [31]. Aoyama et al. [21] found that 1-year freedom from new BMs improved from 41.5% to 63.7% by adding WBRT to SRS treatment. 

However, this measure is accompanied by worse neurocognitive outcomes and diminished quality of life. Chang et al. [22] evaluated neurocognitive function using the Hopkins Verbal Learning Test-Revised (HVLT-R) in a group of 58 patients randomized to SRS or SRS+WBRT. SRS alone was found to provide a substantial neurocognitive benefit. Kocher et al. [8] reported that WBRT added to SRS has a detrimental effect on quality of life and does not improve the median duration of functional independence despite improving local and intracranial control. Brown et al. [23] reported a significant deterioration in immediate recall, delayed recall, and verbal fluency in a large randomized cohort of 213 patients with 1–3 BMs treated with WBRT. All these reports provide evidence that in patients with a limited number of metastases, the risks of adding WBRT to SRS outweigh the benefits. Adjuvant WBRT adds approximately 2 weeks of treatment time, offsetting one of the benefits of SRS in a palliative indication. In addition, WBRT might increase the risk of RN. Despite an increased intracranial control, randomized controlled trials did not show a survival benefit by combining SRS and WBRT [8,21]. 

Of all patients in our study treated with SRS alone, 39.6% later received WBRT for multiple new metastases. The high brain recurrence rate of 52.6% within 1 year demonstrates that while omitting or postponing WBRT has a favorable impact on cognition and quality of life, frequent monitoring for new metastases with MRI is crucial and, therefore, routinely performed at our clinics. 

Our study further confirms that SRS as the single modality to treat solitary or multiple brain lesions is suitable and effective, delivering high local control rates, postponing neurotoxic WBRT, and avoiding invasive surgical intervention. However, in contemporary personalized treatment approaches, the value of histologic and molecular assessment becomes more and more relevant in multidisciplinary decision making. Tumor-specific markers, such as EGFR, BRAF, and HER2, enable targeted treatment options. Surgical resection should be considered alone or in combination with SRS, especially in symptomatic cases or in the case of larger lesions that have a high risk of radiation necrosis after SRS. In addition to achieving rapid symptom relief, in these circumstances, surgical resection also provides tissue for pathological diagnosis and individualized treatment choices. For instance, patients with EGFR mutations have been demonstrated to exhibit better prognoses with brain metastases. This finding has been considered in the updated diagnosis-specific Lung-molGPA score [32]. In our smaller cohort of mixed metastatic histologies specifically treated with SRS, EGFR status did not reveal a significant correlation with survival. However, reports from larger prospective studies have discussed the importance of integrating molecular markers in the prognosis assessment, which can also be presumed relevant in the case of SRS treatment planning [33,34].

Numerous prognostic factors have been proposed in order to improve the estimation of survival, including age, KPS, histology, the number and volume of lesions, the time to appearance of BMs, the status of systemic disease, and the presence of neurologic symptoms and extracranial metastases, as well as response to steroids. According to Kondziolka et al. [35], the prediction of OS in BM patients needs to be improved by considering these prognostic factors. In this study, a survey among physicians was conducted, asking them to estimate individual patient’s survival before SRS treatment based on these parameters. Thus, Kondziolka et al. [35] demonstrated that 49% of physician’s predictions deviated more than 6 months from actual survival, and 18% were off by more than 12 months. This result clearly indicates a continued strong need for more objectifiable assessment criteria to enable treating radiotherapists to communicate more reliable prognoses to their BM patients.

Our study revealed that the presence of extra-cerebral metastases, unfavorable histology, lower KPI, progressive systemic disease, and male sex were significantly associated with shorter survival upon univariate analysis. By multivariate analysis, only lower KPI, progressive systemic disease, and, again, male sex turned out to be significant factors impairing life expectance. No correlation between age and survival could be found, indicating that SRS is a safe and effective treatment option also for elderly patients. This is congruent with the results of Rades et al., who investigated treatment approaches for BMs in elderly patients and confirmed SRS to be a viable option for non-resectable BMs [36]. 

Two negative effects derive from erroneous prognosis assessment. Overestimating survival may lead to overtreatment by subjecting patients with actually poor prognosis to invasive, complicated, and cost-intensive measures. On the other hand, underestimating survival prognosis may lead to inadequate therapy in terms of best supportive care or WBRT only, thereby putting the patient at risk of premature death and long-term cognitive side effects, as well as limiting options for later salvage therapies. Thus, a variety of PIs have been developed in order to better estimate patient survival and to guide physicians in selecting the most suitable therapy for the individual patient. 

Of all the compared indices in our study, the SIR score is the most complex PI considering the largest amount of prognostic factors, and as the only PI, it requires the additional assessment of the volume of the largest metastasis before treatment planning. In our study, however, SIR also turned out to be the only PI not significantly associated with survival. The score tends to unevenly distribute patients between prognostic subgroups, which has already been noticed in previous reports [14]. When applying the SIR score in our cohort, only three patients were attributed to the poor prognosis subgroup, whereas 102 were classified intermediately and 21 as having a good prognosis. In particular, the SIR cut-offs for the largest lesion size (I: 0–5 ccm, II: 5–13 ccm, and III: >13 ccm) may not be suitable to adequately sub-categorize average patients, which might potentially profit from SRS treatment. As evidence, only 1 of our patients exhibited the largest metastasis volume of more than 13 ccm, 16 patients of 5–13 ccm, and 90 of less than 5 ccm. This inherent weakness of the SIR score is likely to be the main cause for the missing correlation with OS evidenced in our study.

The comparatively simple BSBM score showed the strongest correlation with survival and the clearest distinction between prognostic groups. The score was created and validated on patients treated with SRS only. It requires just three parameters (KPS, control of primary tumor, and extracranial metastases) divided into two categories each, all of which can easily be assessed before treatment.

However, OS prediction based on any applied prognostic scoring did not deliver acceptable consistency with the OS monitored in our patient cohort. Even according to the most reliable BSBM score, originally published OS prediction for the classified patient sub-groups differs substantially from our data set. Lorenzoni et al. [16] reported median OS in the four prognostic groups of 1.9, 3.3, 13.1, and >32 months. The actual median OS observed in our study was 3, 10, 18, and 50 months for the corresponding groups. Superior survival might be explained by continuously improved cancer treatment regimens during the last decade. In addition, several indices (RPA, GPA, and that of Rades et al.) were validated for patients primarily treated with WBRT, thereby lacking representation of SRS patients only. In conclusion, more recent median survival data often substantially differ from the original PI studies. This finding indicates that improved OS data of state-of-the-art cancer treatment regimens should finally be considered when presenting OS estimations still performed based on PIs that have been partially established for decades. 

Consistently, in our present study, even the most reliable score classification (BSBM) did not deliver superior OS predictions when compared to physicians just estimating their patient’s survival on the basis of their clinical experience, as reported by Kondziolka et al. [35]. Of the 2700 OS predictions, physicians were able to correctly estimate survival in 51% of cases within ± 6 months. As an analogy, when applying the best predicting BSBM score to our patient cohort, only 46% of OS predictions deviated less than 6 months from the median observed OS in each corresponding prognostic subgroup. Using the same score, a prognostic deviation of more than 12 months was found in 28% of our patients, as compared to the reported 18% physician’s predictive inaccuracy by Kondziolka et al.

However, score reliability might be enhanced by including more recently discovered independent prognostic factors, such as initial brain metastasis velocity (iBMV) [37], also defined by others as distant metastasis velocity (DMV) [38]. Furthermore, the inclusion of histology-specific biomarkers is reported to improve survival estimation. The diagnosis-specific GPA score adds EGFR, BRAF, hemoglobin, HER2, and breast cancer subtype as factors that enhance the scoring accuracy for the respective tumors. Indeed, in our analysis, the updated ds-GPA score markedly improved the correlation with survival in comparison with the original GPA. However, even at the cost of added complexity, it did not outperform the much simpler BSBM score. In addition, female sex has also been shown to be an advantageous prognostic factor in several tumor entities [39,40,41,42]. However, none of the common PIs includes sex as a prognostic factor, although correlations between OS and sex in SRS treatment of BMs have occasionally been reported in the literature [9]. While most scoring systems have been developed to estimate survival rates across all patients with BMs regardless of the treatment method, our study focuses specifically on patients who received SRS alone. Similarly, other research examining comparable patient groups has identified significant survival differences based on sex [5,9,43]. To assess whether sex acts as an independent factor in survival outcomes, we conducted a multivariate analysis that included various primary tumor types. This analysis confirmed that the survival benefit associated with the female sex exists independently of the primary tumor origin. This finding underscores the importance of considering sex in the context of treatment decisions, even as the pharmacogenetic and molecular mechanisms underlying the survival advantages observed in females continue to be explored. These observed differences are nevertheless crucial for clinicians making treatment decisions.

Our results significantly affirm a substantially prolonged OS of female patients versus male patients with a doubled median survival time, as recently reported [44]. This difference might be explained, only partially, by the fact that breast cancer predominantly occurs in women and exhibits a better prognosis when compared to other histologies. However, even when breast cancer patients are excluded from the analysis, females still exhibit a significant survival advantage (15 months versus 9 months in males) after SRS of BMs. In fact, by excluding sex-specific tumor histologies from the analysis, no significant inhomogeneity of known outcome-influencing parameters could be detected, which could explain the female survival advantage over male patients, except for the number of brain metastases. Unexpectedly, however, female patients in our cohort presented an even higher average number of BMs, which is actually considered, by itself, a negative predictor of survival. This finding clearly indicates a yet unknown mechanism directly related to the female sex, which effectively enhances OS after SRS of brain metastasis. 

After further validating and confirming our results in larger studies, sex, and eventually other independent parameters, should finally be included in revised prognostic indices for the treatment of BMs with SRS to enhance the reliability of prognostic scoring.

## 5. Conclusions

SRS of brain metastases of different histological origins is confirmed to represent a safe and effective treatment option, with advantages regarding quality of life and local control, when frequent monitoring of new metastases with MRI is performed. At best, prognostic indices in use are helpful tools to roughly estimate survival. Finally, our findings strongly indicate that sex represents a novel independent prognostic factor for survival after radiosurgery of brain metastases. Consequently, sex should be considered a reliable candidate to be incorporated into revised prognostic indices for cranial SRS treatment. 

## Figures and Tables

**Table 1:** Patient characteristics.

Characteristic		n (%)
Patients	n	108
Number of SRSs	n	132
Number of SRSs per patient	Median	1
	Range	1–5
	Only one SRS in lifetime	82 (75.9%)
Number of metastases	n	213
Number of metastases treated per SRS	Median	1
	Range	1–5
	Only one metastasis treated	75 (56.8%)
Sex	Female	51 (47.2%)
	Male	57 (52.8%)
Age	Median	63
	Range	22–85
Primary tumor	NSCLC	51 (47.2%)
	NSCLC adeno	40 (37%)
	NSCLC squamous cell	9 (8.3%)
	Breast	11 (10.2%)
	Melanoma	20 (18.5%)
	RCC	9 (8.3%)
	Others	19 (17.6%)
Time to brain metastasis [months]	Median	14.5 months
	Range	0–439 months
KPI	<80	24 (18.2%)
	≥80	108 (81.8%)
Immobilization	Stereotactic ring	121 (91.7%)
	Thermoplastic mask	11 (8.3%)
SRS dose (80% isodose)	Median	20 Gy
	Range	13–22 Gy
Volume of largest metastasis	<5 ccm	99 (87.6%)
	5–13	13 (11.5%)
	>13 ccm	1 (0.9%)
Extracranial metastases	Yes	95 (74.8%)
	No	32 (25.2%)
Controlled primary	Yes	59 (46.5%)
	No	68 (53.5%)

**Table 2:** Univariate and multivariate analyses of prognostic factors influencing survival after SRS.

Factor		Univariate		Multivariate	
		OR (95% CI)	*p*-Value	OR (95% CI)	*p*-Value
Age		1.01 (0.99–1.01)	0.489		
Time to metastasis		1.00 (1.00–1.00)	0.844		
Number of metastases		0.88 (0.68–1.13)	ns (0.309)		
Volume of largest metastasis		1.06 (0.97–1.14)	0.188		
Extra cerebral metastases	Present	2.60 (1.56–4.33)	<0.001	1.26 (0.60–2.66)	0.538
	Not present	1.0 (ref)	NA	1.0 (ref)	NA
Dose		1.33 (0.97–1.83)	0.077		
Sex	Female	0.52 (0.34–0.78)	0.001	0.56 (0.34–0.91)	0.020
	Male	1.0 (ref)	NA	1.0 (ref)	NA
Histology	NSCLC adeno	1.0 (ref)	0.018	1.0 (ref)	0.36
	NSCLC squamous	2.16 (0.99–4.71)	0.054	2.42 (1.07–5.47)	0.033
	Breast	0.69 (0.35–1.34)	0.270	1.13 (0.52–2.44)	0.756
	Melanoma	1.02 (0.57–1.82)	0.953	1.41 (0.75–2.67)	0.288
	RCC	1.92 (0.94–3.89)	0.072	1.49 (0.68–3.24)	0.319
	Other	2.16 (1.18–3.94)	0.012	1.54 (0.82–2.90)	0.184
KPI		0.77 (0.67–0.89)	<0.001	0.77 (0.66-0.90)	<0.001
Systemic disease status		1.57 (1.30–1.91)	<0.001	1.45 (1.10–1.92)	0.008
NSCLC EGFR	Mutated	0.66 (0.24–1.712)	0.404		
	Negative or unknown	1.0 (ref)	NA		
Melanoma BRAF	Mutated	0.71 (0.27–1.91)	0.501		
	Negative or unknown	1.0 (ref)	NA		
Breast subtype	Luminal A	1.0 (ref)	NA		
	Luminal B	0.12 ( 0.02–1.32)	0.084		
	Her2-positive	0.06 (0.01–1.13)	0.060		
	Triple-negative	3.75 (0.32–44.41)	0.295		

**Table 3:** Kaplan–Meier analysis of overall survival for different prognostic indices.

Score	Group	Patients in Group (n)	Median OS (Months)	Lower 95% KI	Upper 95% KI	*p*-Value (Log Rank)
Overall OS			15	9.87	20.13	
BSBM	0	14	3	0.00	8.50	<0.0001
	1	53	10	6.21	15.79	
	2	36	18	13.52	22.48	
	3	17	50	40.40	59.60	
GPA	0–1	14	5	4.09	5.91	0.001
	1.5–2.5	89	16	11.16	20.84	
	3	16	15	8.77	21.23	
	3.5–4	7	61	40.92	81.08	
ds-GPA	0–1	11	4	0.00	7.43	<0.0001
	1.5–2.5	7	19	0.05	30.61	
	3	45	20	10.05	19.98	
	3.5–4	30	42	11.39	61.72	
SIR	0–3	3	6	2.80	9.20	0.095
	4–7	102	16	8.72	17.28	
	8–10	21	39	12.74	52.98	
RPA	III	10	5	3.48	6.52	<0.0001
	II	104	15	10.65	19.35	
	I	15	49	41.74	56.26	
Rades et al.	9–10	8	5	3.66	6.34	<0.0001
	11–13	22	12	6.99	17.01	
	14–16	68	13	7.68	18.32	
	17–18	28	49	39.54	58.46	

## Data Availability

The data presented in this study are available upon request from the corresponding author.
